# Exploring the Effect of the Dynamics of Behavioral Phenotypes on Health Outcomes in an mHealth Intervention for Childhood Obesity: Longitudinal Observational Study

**DOI:** 10.2196/45407

**Published:** 2023-08-17

**Authors:** Sarah Woo, Sunho Jung, Hyunjung Lim, YoonMyung Kim, Kyung Hee Park

**Affiliations:** 1 Department of Medical Sciences College of Medicine Hallym University Chuncheon-si Republic of Korea; 2 School of Management Kyung Hee University Seoul Republic of Korea; 3 Department of Medical Nutrition Kyung Hee University Yongin-si Republic of Korea; 4 University College Yonsei University International Campus Incheon Republic of Korea; 5 Department of Family Medicine Hallym University Sacred Heart Hospital Hallym University Anyang-si, Gyeonggi-do Republic of Korea

**Keywords:** behavioral dynamics, behavioral phenotype, functional data analysis, FDA, machine learning analysis, mobile health, mHealth, obesity intervention, pediatric obesity, mobile phone

## Abstract

**Background:**

Advancements in mobile health technologies and machine learning approaches have expanded the framework of behavioral phenotypes in obesity treatment to explore the dynamics of temporal changes.

**Objective:**

This study aimed to investigate the dynamics of behavioral changes during obesity intervention and identify behavioral phenotypes associated with weight change using a hybrid machine learning approach.

**Methods:**

In total, 88 children and adolescents (ages 8-16 years; 62/88, 71% male) with age- and sex-specific BMI ≥85th percentile participated in the study. Behavioral phenotypes were identified using a hybrid 2-stage procedure based on the temporal dynamics of adherence to the 5 behavioral goals during the intervention. Functional principal component analysis was used to determine behavioral phenotypes by extracting principal component factors from the functional data of each participant. Elastic net regression was used to investigate the association between behavioral phenotypes and weight change.

**Results:**

Functional principal component analysis identified 2 distinctive behavioral phenotypes, which were named the *high or low adherence level* and *late or early behavior change*. The first phenotype explained 47% to 69% of each factor, whereas the second phenotype explained 11% to 17% of the total behavioral dynamics. *High or low adherence level* was associated with weight change for adherence to screen time (β=−.0766, 95% CI −.1245 to −.0312), fruit and vegetable intake (β=.1770, 95% CI .0642-.2561), exercise (β=−.0711, 95% CI −.0892 to −.0363), drinking water (β=−.0203, 95% CI −.0218 to −.0123), and sleep duration. *Late or early behavioral changes* were significantly associated with weight loss for changes in screen time (β=.0440, 95% CI .0186-.0550), fruit and vegetable intake (β=−.1177, 95% CI −.1441 to −.0680), and sleep duration (β=−.0991, 95% CI −.1254 to −.0597).

**Conclusions:**

Overall level of adherence, or the *high or low adherence level*, and a gradual improvement or deterioration in health-related behaviors, or the *late or early behavior change*, were differently associated with weight loss for distinctive obesity-related lifestyle behaviors. A large proportion of health-related behaviors remained stable throughout the intervention, which indicates that health care professionals should closely monitor changes made during the early stages of the intervention.

**Trial Registration:**

Clinical Research Information Science KCT0004137; https://tinyurl.com/ytxr83ay

## Introduction

### Background

Interest in behavioral phenotypes has been growing rapidly in the field of chronic lifestyle diseases, such as obesity, over the last decade as precision medicine is undergoing massive development [1]. The term *behavioral phenotype* refers to social, cognitive, and behavioral characteristics associated with a genetic or physiological disease [2]. Within the context of obesity, it refers to the individualized behavioral patterns associated with obesity because of the interaction between heredity and the environment, including food-seeking tendencies and reward-sensitive traits [3,4]. In this context, behavioral phenotypes allow a better understanding of the individual characteristics relevant to the development and treatment of obesity [5].

Previous studies have mainly described the aspects of behavioral phenotypes based on the characteristics determined before the intervention [4-6]. However, the framework of *behavioral phenotype* can be further expanded to account for the dynamics of behavioral phenotype during the intervention [1], as there may be considerable heterogeneity in behavioral changes across people and time [7]. The concept of behavioral dynamics, which describes the time evolution of behavioral phenotypes observed during an intervention, has recently attracted much attention in the area of mobile health (mHealth). Behavioral changes, such as enhanced healthy dietary habits and physical activity, are known to be associated with weight changes after obesity intervention [8,9]. Nonetheless, the potential role of behavioral dynamics in obesity intervention remains largely unexplored, partly because of the lack of a reliable statistical technique suitable for measuring and analyzing behavioral evolution patterns [10-12].

To an extent, the measurement of such behavioral change phenotypes has become possible with developments in mHealth technologies. The features of mHealth enable greater accessibility to provide feedback regarding adherence to behavioral goals via push notifications, text messages, phone calls, and video meetings [13-16]. In addition, to provide real-time feedback, mHealth technologies can be used to collect data associated with treatment outcomes such as behavioral changes during the intervention [17]. Advancements in mHealth provide a useful tool for monitoring behavioral changes during an intervention, thus enabling the collection of time-series data with latent dynamic patterns [18]. These data can be used to explore behavioral dynamics using appropriate analysis models to handle temporal dynamics. As summary statistics, such as average over time, are unable to address behavioral dynamics during the intervention, this study proposes a method to reveal the pattern of behavioral change over time in a behavioral time series.

### Objectives

Hence, this study intended to model behavioral dynamics and show how the temporal evolution of behavioral phenotypes can be used to predict weight changes following an mHealth intervention for children and adolescents with overweight or obesity through a hybrid approach combining functional data analysis (FDA) and machine learning analysis [19].

## Methods

### Recruitment

A total of 160 children and adolescents aged 8 to 16 years completed the Intervention for Childhood and Adolescent Obesity via the Activity and Nutrition-Online study between May 2020 and June 2021. Overall, 64 participants with missing data for more than 20% of the behavioral phenotype variables were excluded from the data analysis [20]. The final sample of 88 participants (62/88, 71% male), with the first and last observations for all variables, was included in the analysis. For data imputation, linear interpolation, a nonparametric method for estimating a curve, was used to fill in the missing data [21].

The Intervention for Childhood and Adolescent Obesity via the Activity and Nutrition-Online study was a single-arm trial aimed at investigating the feasibility and effectiveness of an mHealth intervention to treat children and adolescents with overweight or obesity during the COVID-19 pandemic, using video meetings and individual chat rooms as the primary mHealth platform. Because of the pandemic, children and adolescents were expected to comply with the “social distancing in daily life” policy (2020 to 2021) during the intervention phase. The policy included sporadic restrictions on school attendance and the use of public facilities, which may have impacted children’s health-related behaviors. This study was conducted at a university hospital in South Korea, and participants were recruited through web-based advertisements and invitation letters sent to schools. Children and adolescents with an age- and sex-specific BMI ≥85th percentile, according to the 2017 Korean National Growth Chart [22], were eligible for participation. Participants taking medications that affected weight, including steroids and insulin, were excluded from the study.

### Ethics Approval

The study was conducted in accordance with the guidelines specified in the Declaration of Helsinki, and the study protocol was approved by the institutional review board of Hallym University Sacred Heart Hospital (HALLYM 2019-04-027-005).

### Informed Consent and Trial Registration

Written informed consent was obtained from all participants and their primary caregivers. The trial was registered at Clinical Research Information Science [23] (KCT0004137).

### Intervention

The multidisciplinary mHealth intervention was a 6-month trial consisting of 1:1 behavior modification and nutrition counseling with exercise education. Physical examinations and doctoral consultations were conducted during the hospital visit and individual goals were set accordingly. After the initial examination, intervention was performed using a mobile platform.

The behavior modification and nutrition counseling sessions, each lasting 30 minutes, were alternated on a weekly basis. Overall, 10 behavior modification counseling sessions and 8 nutrition counseling sessions were provided to the children and adolescents. Each counseling session was held using Zoom (Zoom Video Communications), a videoconferencing tool.

Exercise educational materials were provided weekly through an individual KakaoTalk chat room (Kakao Corp), a widely used mobile messaging app in South Korea. Audiovisual exercise materials were provided using the Intervention for Childhood and Adolescent Obesity via the Activity and Nutrition exercise app developed by the researchers. Physical activity was monitored with a step-tracking watch (Seven Elec Co) and individual feedback messages were provided through a mobile app.

### Measurement

#### Anthropometric Measurement

Anthropometric measurements were conducted at baseline and 6-month follow-up. Height and weight were measured to the nearest 0.1 cm and 0.1 kg, respectively, with a stadiometer and bioelectrical impedance analysis (InBody 720 Body Composition Analyzer, BioSpace Co., Ltd). Furthermore, BMI was calculated as weight (kg) divided by height squared (m^2^), and the BMI *z* score was computed according to age- and sex-specific BMI using the 2017 Korean National Growth Chart [22]. The BMI *z* score delta value, the primary outcome of the study, was calculated as the post-BMI *z* score minus the pre-BMI *z* score.

#### Questionnaires

Demographic information, including age, sex, monthly household income, and parental BMI, was collected using a self-reporting questionnaire answered by the primary caregivers. Lifestyle variables, including weekday and weekend sleep duration (eg, “During the past week, what time did you go to bed and wake up?”), frequency of daily physical activity (“How frequently do you exercise each day, including school and afterschool activities?”), frequency of daily screen time (eg, “During the past week, how much time did you spend time watching TV, using smartphone, tablets, or computer each day?”), and the frequency of weekly vegetable and beverage consumption (eg, “During the past week, how many servings of fruits and vegetables did you eat?”), were also collected using a questionnaire reported by children and adolescents.

The stages of change were assessed using a question derived from the transtheoretical model of change [24], where children and adolescents responded to the readiness of changing weight, physical activity, and dietary behaviors on a 5-point scale (Precontemplation=“Currently, I am not engaged in changing the [target behavior] and do not attempt to try to change the [target behavior] in the following six months”; Contemplation=“Currently, I am not engaged in changing the [target behavior], but I am willing to try to change the [target behavior] in the following six months”; Preparation=“Currently, I am not engaged in changing the [target behavior], but I plan to change the [target behavior] in the following 30 days”; Action=“I am currently doing the [target behavior], and I have started doing the [target behavior] in the past six months”; and Maintenance=“I am currently doing the (target behavior), and I have been doing the [target behavior] for more than six months”).

#### Psychological Variables

The child’s self-esteem was measured using the Rosenberg Self-esteem Scale validated in Korean [25,26], which comprises 10 items and uses a 5-point Likert scale. A higher score indicates higher self-esteem, and the reliability of the scale is reported as 0.75 to 0.87.

The children’s problem behaviors were measured using the Child Behavior Checklist 6-18, which was developed by Achenbach [27] and validated in Korean [28]. The Child Behavior Checklist 6-18, using a Likert scale from 0 to 2, consists of 86 items responded to by the child’s caretaker. The subscales for problem behaviors included internalizing and externalizing problems, which refer to a group of psychological symptoms and behaviors that are turned toward the individual (eg, depressive or anxious symptoms) or expressed against the external environment (eg, aggressive behavior or attention problems). The raw scores were standardized to T scores (mean 50, SD 10), and T scores of ≥64 and ≥ 60 indicate clinical and subclinical ranges, respectively.

The Psychological Well-Being Index-Short Form was used to measure the parental psychological stress. The scale has 18 items assessed using a 4-point Likert scale, which was developed and standardized based on the General Health Questionnaire [29,30]. An example of the questionnaire’s item is “Nowadays, I feel very comfortable and healthy,” and a higher score indicates a higher level of stress.

The Korean-Parents as Social Context Questionnaire was used to assess parenting behaviors. The Korean-Parents as Social Context Questionnaire, developed by Skinner et al [31] and validated in Korean by Jung and Shin [32], comprises 23 questions and is measured using a 4-point Likert scale. The positive parenting behaviors comprise “warmth,” “structure,” and “autonomy support” subscales, and the negative parenting behaviors are constituted of “rejection,” “chaos,” and “coercion” subscales.

#### Behavioral Phenotypes

Behavioral phenotypes were determined based on the temporal dynamics of adherence to behavioral goals during the intervention, named “mission 5.” “Mission 5” comprises 5 daily behavior goals associated with weight change, which were selected based on clinical practice guidelines for treating pediatric obesity [33]: screen time of <2 hours (mission 1) [33], eating >5 servings of fruits and vegetables (mission 2) [34,35], exercising >1 hour (mission 3) [33], drinking water or plain milk instead of sugar-sweetened beverages (mission 4) [33], and sleeping more than >8 hours (mission 5) [35]. Compliance with each behavioral goal during the past week was measured on a 5-point Likert scale biweekly, which was monitored at every behavioral modification counseling session. The behavioral phenotypes that corresponded to the changing dynamics of each goal were estimated using functional principal component analysis (FPCA) and were used as predictors for weight change.

#### Procedures

The main feature of this study’s approach is that we used a hybrid 2-stage procedure of FPCA and elastic net regression to predict weight changes based on behavioral phenotypes during an mHealth intervention. The first stage of the approach uses FPCA to detect the key shapes of the underlying patterns of behavioral change. The second stage of the approach applies elastic net regression [36], a machine learning–based regression model for predicting weight change, which is based on the functional principal component (PC) scores derived from the first stage, along with a variety of obesity-related covariates.

**Figure 1:**
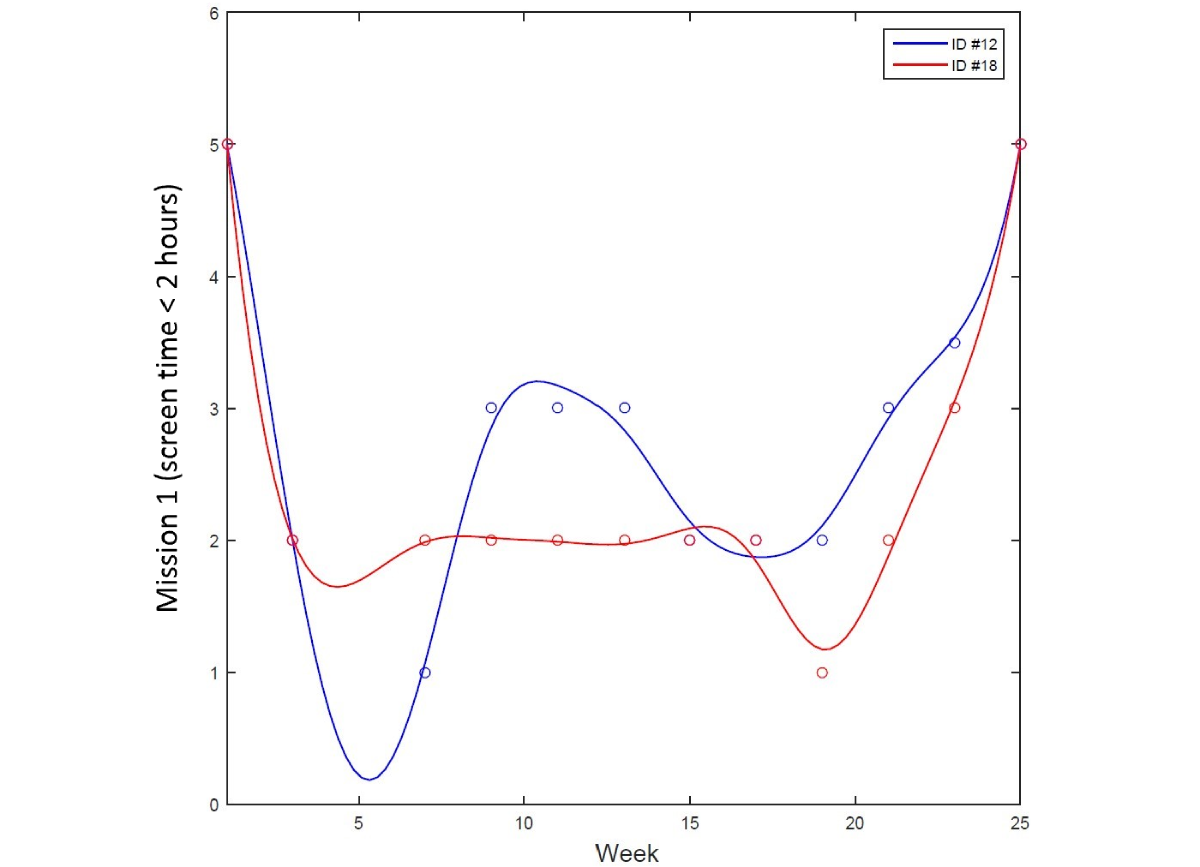
Example of dynamics of behavioral change during a mobile health intervention.

**Figure 2:**
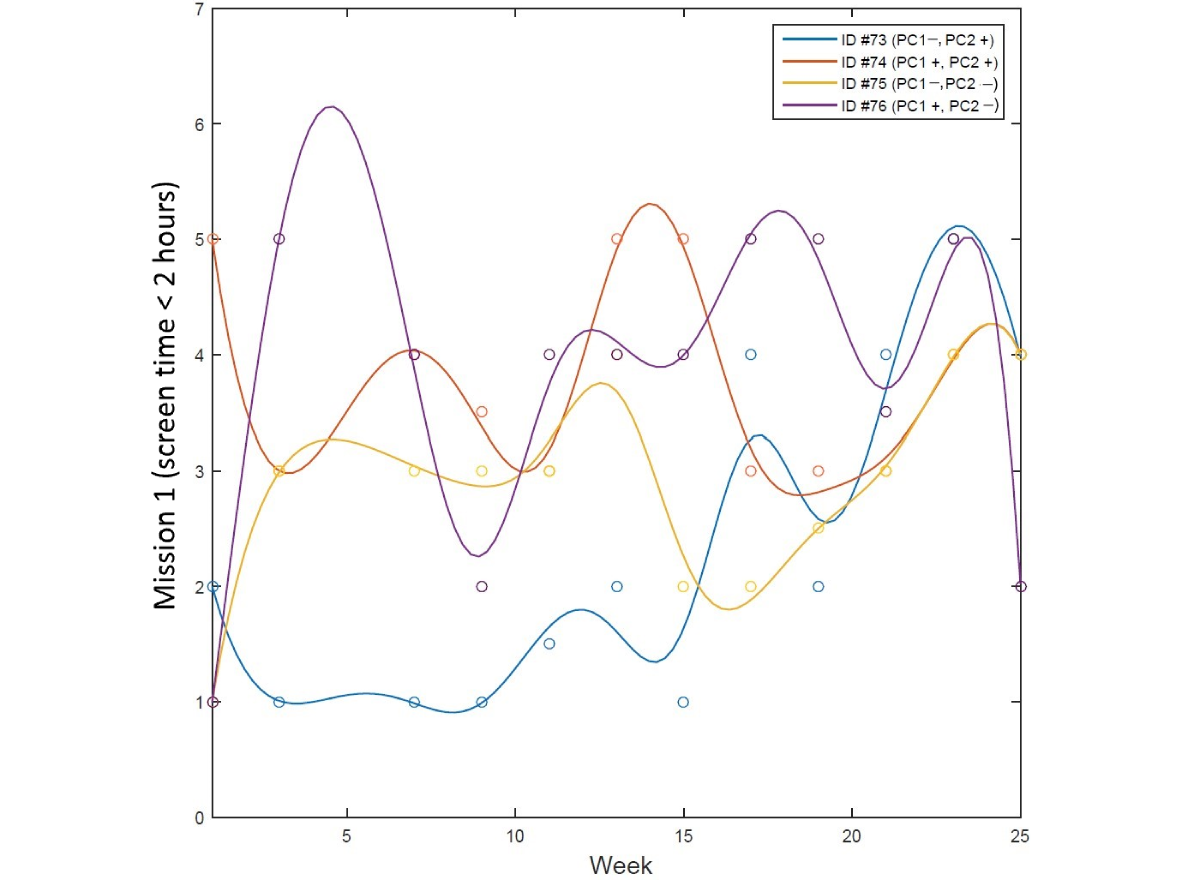
Smoothing splines of each participant’s behavioral dynamics (mission 1). PC: principal component.

We used the FDA framework, as it allows the prediction of intervention outcomes using time-varying behavioral data, whereas conventional analytical methods handle data points collected from a certain time point [19]. The longitudinal data observed from each participant were expressed as a function, represented as a single unit of observation, and unaffected by the assumptions of correlations between measurements [37]. Then, each functional profile was represented as a smooth curve estimated from the discrete data using a smoothing technique to uncover the latent curve. Figure 1 illustrates an example of longitudinal behavioral dynamics during the intervention, expressed as a single unit of functional data. Each functional data point shows a distinctive pattern of changing dynamics during the course of intervention, even when they have the same observed scores at weeks 1 and 25. As the FDA is a data-driven, nonparametric method that is highly flexible in treating nonequally measured time-series data [19,37], it was used in this study to assess behavioral dynamics. Figure 2 shows an example of the smoothing process for the splines of each participant. Each line in distinctive colors indicates the dynamics of each participant, wherein the data points observed at discrete time points, shown as circles, are represented as a single function using the smoothing technique.

The PCs were then derived from the functional data using FPCA. The FPCA is a frequently used method to reduce the dimensions of functional data obtained from the FDA, which identifies the representative dynamic profiles of individual functions. Using FPCA, functional data were transformed into PCs, and functional PC scores were derived for a clearer interpretation of the dynamic patterns during the first stage [19,38].

Elastic net regression, the second part of the hybrid 2-stage approach, is a machine learning–based regression method for building a predictive model with high-dimensional low-sample size data [36]. The elastic net combines the penalization techniques of ridge and lasso regression, which uses both continuous shrinkage and automatic variable selection and has a higher prediction accuracy than ridge or lasso regression separately. In the 2-step approach, the functional PC scores from the first stage were included in the elastic net model as regressors to investigate the link between the dynamic patterns of behavioral phenotypes and the health outcomes of obesity intervention.

### Statistical Analysis

Baseline characteristics were presented as mean (SD). The Shapiro-Wilk test was used to test normality, and variables that were nonnormative were log-transformed, and geometric means were shown. Penalized smoothing splines were used to convert the longitudinal data into smooth curves. The PCs were derived using FPCA by reducing the dimensions of the functional variables. The cutoff point for selecting the total number of PCs retained for each behavioral phenotype was 70% of the total variability [39]. Subsequently, a k-means cluster analysis was conducted to determine the clusters according to the PC dimensions.

For the elastic net model, the variables were standardized to compare the sizes of the coefficients. The regularization parameters of the elastic net regressions were optimally chosen via 10-fold cross-validation. First, α was estimated as .01, and the following steps were repeated 1000 times: (1) running cross-validation to determine the value of lambda that gives the minimum mean cross-validated error and (2) extracting the regression coefficients using the optimal value of lambda. The log-lambda value that showed the minimum mean cross-validated error was estimated as 0.8380. Finally, bootstrap samples were used to estimate the covariate stability of the model to establish the stability of the parameters. The median regression coefficients across the 500 bootstrap samples were computed unless a predictor failed to survive more than 10% of the 1000 repetitions with a nonzero coefficient. Statistical significance was determined at *P*<.05, and analyses were conducted with MATLAB (R2015a) using the *glmnet* package.

## Results

### Baseline Characteristics

Baseline characteristics of the study participants are presented in Table 1. The mean age of the participants was 11.46 (SD 1.69) years, and 71% (62/88) were male. The baseline BMI *z* score was 2.60 (SD 0.90). Male participants (11.70, SD 1.62) were older than female participants (10.90, SD 1.76), and there were no other significant differences between male and female participants. Among the scores of missions 1 to 5, adherence to exercise for >1 hour per day (4.03, SD 1.39) was the highest, whereas adherence to sleep for >8 hours per day (2.39, SD 1.78) was lowest at baseline.

**Table 1:** Baseline characteristics of participants.

Variables		All participants (n=88)	Male participants (n=62)	Female participants (n=26)	*P* value
Age (years), mean (SD)		11.46 (1.69)	11.70 (1.62)	10.90 (1.76)	.04
Baseline BMI *z* score		2.60 (0.90)	2.67 (0.83)	2.44 (1.04)	.19
**Monthly household income (KRW^a^), n (%)**					.22
	Lowest (<3 million [<US $2331])	7 (8)	3 (5)	4 (15)	
	Middle (3-6 million [US $2331-US $4662])	44 (50)	33 (53)	11 (42)	
	Highest (>6 million [>US $4662])	37 (42)	26 (42)	11 (42)	
Self-esteem^b^, mean (SD)		36.45 (1.27)	37.47 (1.23)	34.15 (1.34)	.15^c^
Internalizing problem^b^, mean (SD)		53.80 (1.22)	53.47 (1.23)	54.62 (1.21)	.65^c^
Externalizing problem^d^, mean (SD)		52.90 (1.21)	52.18 (1.20)	54.65 (1.24)	.31^e^
Stages of change^d^, mean (SD)		3.33 (1.29)	3.30 (1.32)	3.42 (1.21)	.54^e^
Beverage intake^d^ (frequency/wk), mean (SD)		1.78 (1.67)	1.88 (1.69)	1.56 (1.58)	.12^e^
Vegetable intake^d^ (frequency/wk), mean (SD)		3.59 (1.70)	3.65 (1.71)	3.45 (1.69)	.66^e^
Sleep duration (min/d), mean (SD)		537.72 (68.29)	535.35 (67.81)	543.37 (70.45)	.62
Screen time (h/d)^d^, mean (SD)		3.67 (1.51)	3.61 (1.55)	3.82 (1.41)	.54^e^
Exercise frequency (wk)^d^, mean (SD)		2.99 (1.54)	3.01 (1.56)	2.93 (1.50)	.79^e^
Maternal BMI (kg/m^2^)^d^, mean (SD)		24.68 (1.18)	24.67 (1.19)	24.70 (1.15)	.97^e^
Paternal BMI (kg/m^2^), mean (SD)		27.33 (3.13)	27.34 (3.37)	27.30 (2.54)	.96
Maternal psychosocial stress^b^, mean (SD)		17.23 (7.68)	16.21 (7.09)	19.65 (8.60)	.05
Paternal psychosocial stress^b^, mean (SD)		16.73 (7.64)	16.85 (7.59)	16.42 (7.90)	.81
Maternal positive parenting^c^, mean (SD)		27.74 (3.89)	28.26 (3.79)	26.50 (3.93)	.05
Paternal positive parenting^c^, mean (SD)		25.49 (4.34)	25.27 (4.59)	26.00 (3.70)	.48
Maternal negative parenting^c^, mean (SD)		27.01 (7.56)	26.15 (7.68)	29.08 (6.98)	.10
Paternal negative parenting^c^, mean (SD)		25.98 (6.07)	25.85 (5.89)	26.27 (6.58)	.77
Mission 1 (baseline)^d,f^, mean (SD)		2.68 (1.81)	2.65 (1.79)	2.75 (1.86)	.79^e^
Mission 2 (baseline)^d,f^, mean (SD)		3.07 (1.58)	2.98 (1.55)	3.32 (1.66)	.30^e^
Mission 3 (baseline)^d,f^, mean (SD)		4.03 (1.39)	4.18 (1.30)	3.68 (1.57)	.19^e^
Mission 4 (baseline)^d,f^, mean (SD)		3.77 (1.45)	3.74 (1.43)	3.84 (1.49)	.76^e^
Mission 5 (baseline)^d,f^, mean (SD)		2.39 (1.78)	2.47 (1.79)	2.20 (1.78)	.39^e^

^a^KRW: Korean Republic Won.

^b^Parental psychosocial stress was measured using the Psychological Well-Being Index-Short Form (PWI-SF).

^c^Positive and negative parenting styles were measured using the Korean-Parents as Social Context Questionnaire (K-PSCQ).

^d^Geometric mean (SD) presented for nonnormative data.

^e^*P* value of 2-tailed *t* test on log-transformed values.

^f^Mission 1: screen time less than 2 hours; mission 2: eating more than 5 servings of fruits and. vegetables; mission 3=exercising for more than one hour; mission 4=drinking water or plain milk; mission 5=sleeping for more than 8 hours. Data are presented as mean (SD) with the *P* value of the 2-tailed *t* test for continuous variables and as number (%) with the *P* value of the chi-square test for categorical variables.

### FPCA Results

**Figure 3:**
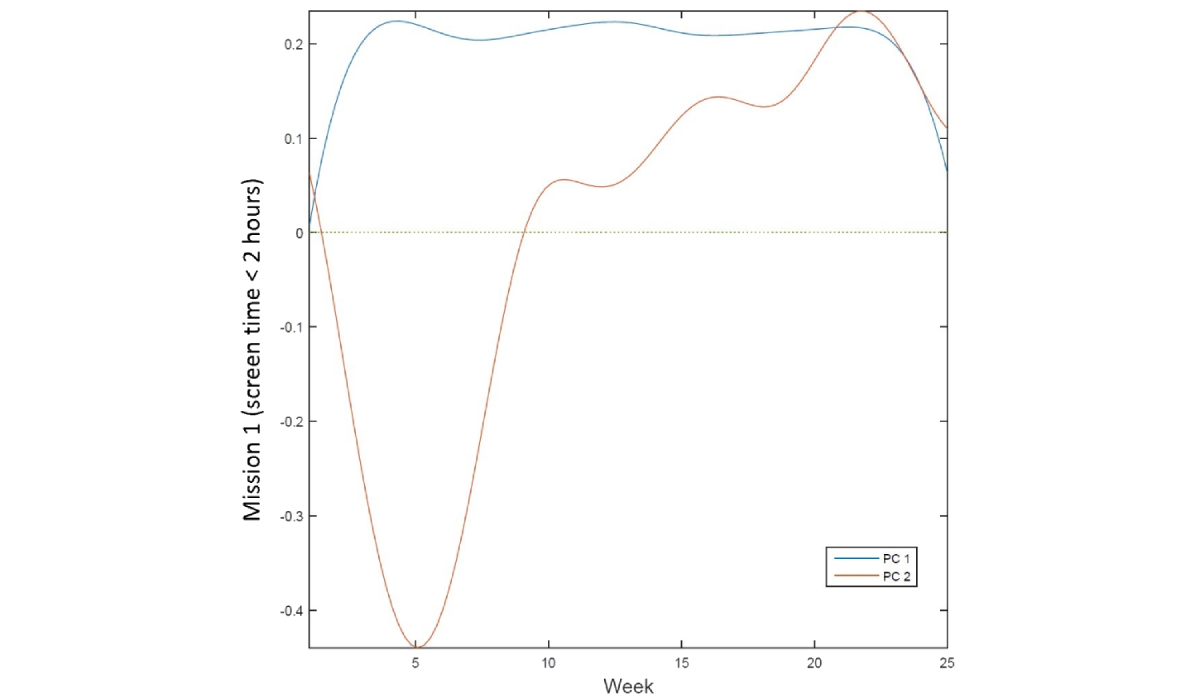
Functional PC curve of Mission 1. PC 1=high or low adherence level; PC 2=late or early behavioral change. PC: principal component.

Figure 3 provides an example of the functional PC curve of mission 1. Participants with a positive first PC score showed a pattern of an overall high level of adherence throughout the intervention, whereas those with a negative score maintained a below-average level of adherence. Thus, the first PC was named as the *high or low adherence level*. The positive score on the second PC showed a pattern of lower adherence at the beginning of the intervention, with an increased level of adherence during the later phase. A negative score represents an opposite pattern of earlier behavior change with lowered participation during the later phase of the intervention. Thus, the second PC was named as the *late or early behavior change*.

**Figure 4:**
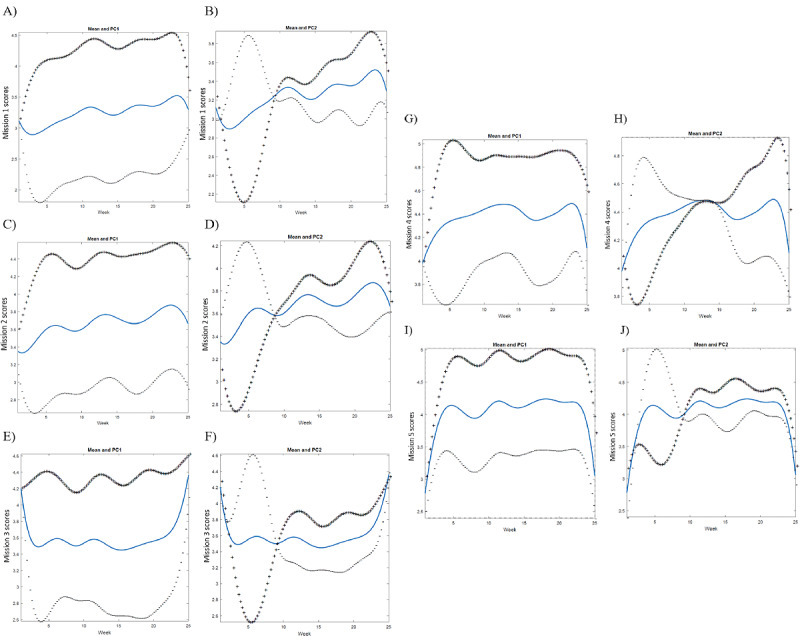
Functional PC curves of the behavioral phenotypes (mission 1-5): mission 1=screen time less than two hours; mission 2=eating more than five servings of fruits and vegetables; mission 3=exercising for more than one hour; mission 4=drinking water or plain milk; mission 5=sleeping for more than eight hours. PC 1=high or low adherence level; PC 2=late or early behavioral change. (A) Mission 1, PC 1, 69% of variance explained. (B) Mission 1, PC 2, 11% of variance explained. (C) Mission 2, PC 1, 60% of variance explained. (D) Mission 2, PC 2, 13% of variance explained. (E) Mission 3, PC 1, 48% of variance explained. (F) Mission 3, PC 2, 17% of variance explained. (G) Mission 4, PC 1, 47% of variance explained. (H) Mission 4, PC 2, 15% of variance explained. (I) Mission 5, PC 1, 52% of variance explained. (J) Mission 5, PC 2, 14% of variance explained. Lines indicate the mean value of all participants (in blue) and the mean ±1 SD of each PC curve (plus and minus signs). PC: principal component.

Figure 4 depicts the high or low scores of the functional PC curves. The curves were visualized to capture the pattern of participants with high or negative scores for each PC, which was represented as the mean value and the mean +1 or −1 SD of each PC score. In PC1, adherence to missions 1, 4, and 5 decreased at the end of the intervention (Figure 4A, 4G, and 4I), but compliance to missions 2 and 3 maintained high scores throughout the intervention (Figure 4C and 4E). Participants with –1 SD from the average had a low score at the beginning of the intervention and maintained a low level below average throughout the treatment.

Participants with +1 SD from the average on PC 2 of behavioral phenotypes 1, 2, 3, and 5 showed a pattern of a low score at the beginning of the treatment, followed by a rapid increase at 8 to 10 weeks of the intervention (Figure 4B, 4D, 4F, and 4J). The scores were above the average after 10 weeks and were maintained throughout the rest of the intervention period. Conversely, participants with −1 SD had a high score at the beginning of the intervention, showed a sharp decrease after 8 to 10 weeks, and remained below average. PC 2 of the fourth behavioral phenotype exhibited a slightly distinctive pattern, wherein participants with +1 SD showed a gradual increase throughout the intervention. In addition, the crossing point of participants with +1 SD and −1 SD was between 10 and 15 weeks of intervention, which is a later point compared with other phenotypes (Figure 4H). The crossing points of participants with average scores of +1 SD and −1 SD from the mean of each PC indicate that a single summary statistic, for example, an average score, cannot illustrate the course of changes at several time points. Even though the overall PC score may be lower, there may be certain periods in which participants with an overall lower PC score overtake their higher counterparts.

### Elastic Net Results

**Figure 5:**
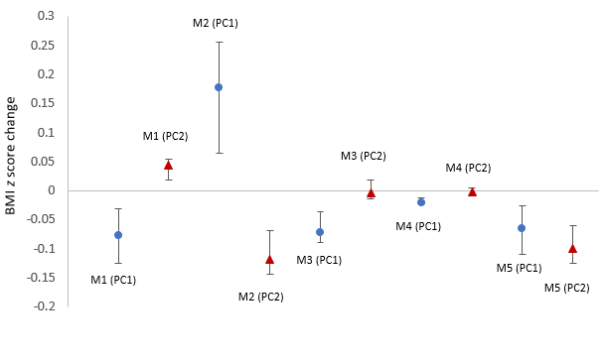
Effect of behavioral phenotypes on BMI z score change. PC: principal component. M1: screen time less than 2 hours; M2: eating more than 5 servings of fruits and vegetables; M3: exercising for more than 1 hour; M4: drinking water or plain milk; M5: sleeping for more than 8 hours; PC 1=high or low adherence level; PC 2=late or early behavioral change. Data were presented as standardized β coefficients and standard regression coefficients with 95% CIs. M: mission.

Table 2 and Figure 5 presents the results of the elastic net regression analysis. After adjusting for confounding variables, the first PC of mission 1 (β=−.0766, 95% CI −.1245 to −.0312), the second PC of mission 2 (β=−.1177, 95% CI −.1441 to −.0680), the first PC of mission 3 (β=−.0711, 95% CI −.0892 to −.0363), the first PC of mission 4 (β=−.0203, 95% CI −.0218 to −.0123), and the first (β=−.0649, 95% CI −.1090 to −.0260) and second PC of mission 5 (β=−.0991, 95% CI −.1254 to −.0597) were negatively associated with an increase in the BMI *z* score. The second PC of mission 1 (β=.0440, 95% CI .0186-.0550]) and the first PC of mission 2 (β=.1770, 95% CI .0642-.2561) were positively associated with increases in the BMI *z* score during the intervention. The elastic net model was adjusted for age, sex, monthly household income, baseline BMI z- score, self-esteem, internalizing problems, externalizing problems, stages of change, beverage intake, vegetable intake, sleep duration, screen time, exercise frequency, attendance rate, maternal BMI, paternal BMI, maternal psychosocial stress, paternal psychosocial stress, maternal positive parenting, paternal positive parenting, maternal negative parenting, and paternal negative parenting.

**Table 2:** Effect of behavioral dynamics on BMI z score change according to elastic net regression model^a^.

Predictors	Adjusted B, unstandardized coefficient (95% CI)^b^	Adjusted β, standardized coefficient (95% CI)^b^
Mission 1: screen time (PC^c^ 1)^d,e^	−0.0031 (−0.0126 to −0.0028)	−.0766 (−.1245 to −.0312)
Mission 1: screen time (PC 2)^d,e^	0.0043 (0.0037 to 0.0131)	.0440 (.0186 to .0550)
Mission 2: fruit and vegetable intake (PC 1)^d,e^	0.0087 (0.0074 to 0.0358)	.1770 (.0642 to .2561)
Mission 2: fruit and vegetable intake (PC 2)^d,e^	−0.0190 (−0.0410 to −0.0175)	−.1177 (−.1441 to −.0680)
Mission 3: exercise (PC 1)^d,e^	−0.0048 (−0.0120 to −0.0044)	−.0711 (−.0892 to −.0363)
Mission 3: exercise (PC 2)^b,c^	−0.0028 (−0.0030 to 0.0041)	−.0038 (−.0146 to .0189)
Mission 4: water intake (PC 1)^b,c^	−0.0039 (−0.0044 to −0.0024)	−.0203 (−.0218 to −.0123)
Mission 4: water intake (PC 2)^b,c^	−0.0032 (−0.0032 to 0.0018)	−.0027 (−.0093 to .0048)
Mission 5: sleep duration (PC 1)^b,c^	−0.0036 (−0.0154 to −0.0033)	−.0649 (−.1090 to −.0260)
Mission 5: sleep duration (PC 2)^b,c^	−0.0146 (−0.0311 to −0.0136)	−.0991 (−.1254 to −.0597)

^a^The model was adjusted for age, sex, monthly household income, baseline BMI *z* score, self-esteem, internalizing problems, externalizing problems, stages of change, beverage intake, vegetable intake, sleep duration, screen time, exercise frequency, attendance rate, maternal BMI, paternal BMI, maternal psychosocial stress, paternal psychosocial stress, Maternal positive parenting included paternal positive parenting, maternal negative parenting, and paternal negative parenting. Regression coefficients for the modeled covariates are not shown.

^b^Data were presented as unstandardized regression coefficients and standard regression coefficients with 95% Cis. The median values are presented as standard regression coefficients.

^c^PC: principal component.

^d^Mission 1: screen time less than 2 hours; mission 2: eating more than 5 servings of fruits and. vegetables; mission 3=exercising for more than one hour; mission 4=drinking water or plain milk; mission 5=sleeping for more than 8 hours.

^e^PC 1: high or low adherence level; PC 2: late or early behavior. change.

### Cluster Analysis Results

**Figure 6:**
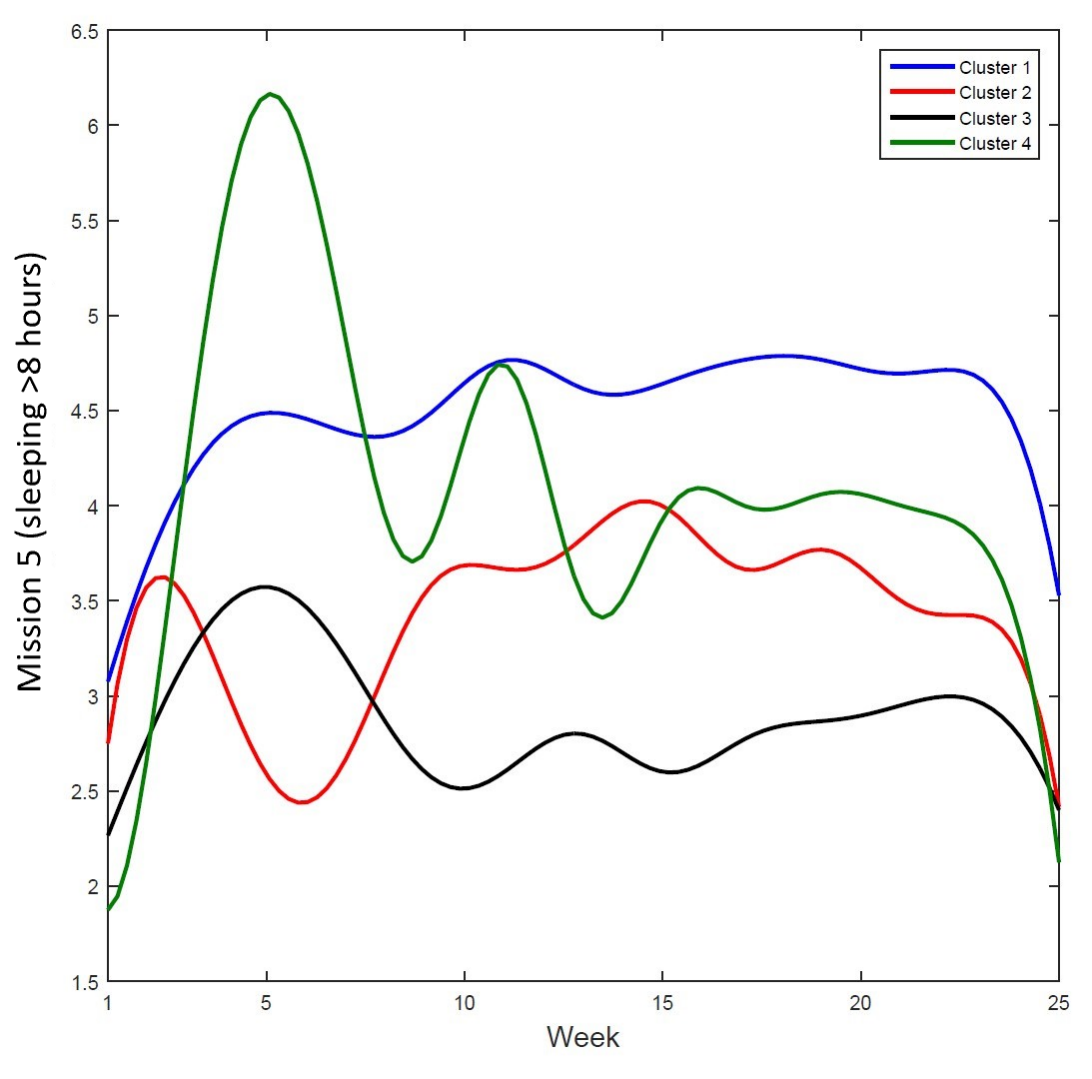
Adherence curves of each cluster PC 1=high or low adherence level; PC 2=late or early behavioral change. Cluster 1: high in PC 1, high in PC 2; cluster 2: low in PC 1, high in PC 2; cluster 3: low in PC 1, low in PC 2; cluster 4: high in PC 1, low in PC2. PC: principal component.

Table 3 shows the results of the k-means cluster analysis for mission 5 (sleep duration). The cluster analysis results of missions 1 to 4 were not included because they showed similar patterns to the presented results. The results showed 4 clusters, a 2 × 2 combination of high or low levels of PC 1 and PC 2. Cluster 1 had a positive value in both PC dimensions, cluster 2 had a negative value in PC 1 and a positive value in PC 2, cluster 3 had a negative value in both dimensions, and cluster 4 had a positive value in PC 1 and a negative value in PC 2. Figure 6 shows the adherence patterns for each cluster throughout the course of the intervention.

Table 4 shows the differences in the baseline characteristics of each cluster of mission 5. The ratio of children was higher in cluster 1 (39/53, 74%), whereas the ratio of adolescents was higher in clusters 2 to 4. The proportion of male participants (10/12, 83%) and participants with lower-than-moderate obesity (9/12, 75%) was the highest in cluster 2.

**Table 3:** Results of k-means cluster analysis of mission 5 (sleep duration)^a^.

	Cluster 1 (n=53)	Cluster 2 (n=12)	Cluster 3 (n=15)	Cluster 4 (n=8)
PC^b^ 1 (high or low adherence level)	2.24	−3.19	−5.71	0.64
PC 2 (late or early behavior change)	0.49	1.78	−1.37	−3.33

^a^Mean value of each PC presented. Cluster 1: high in PC 1, high in PC 2; Cluster 2: low in PC 1, high in PC 2; Cluster 3: low in PC 1, low in PC 2; Cluster 4: high in PC 1, low in PC 2.

^b^PC: principal component.

**Table 4:** Baseline characteristics for clusters of mission 5 (sleep duration).

Variables			Cluster 1 (n=53)^a,b^		Cluster 2 (n=12)^b,c^		Cluster 3 (n=15)^b,d^		Cluster 4 (n=8)^b,e^
**Age group, n (%)**									
	Children (<12 years)	39 (74)		2 (17)		2 (13)		3 (38)	
	Adolescent (≥12 years)	14 (26)		10 (83)		13 (87)		5 (62)	
**Sex, n (%)**									
	Male	37 (70)		10 (83)		9 (60)		7 (75)	
	Female	16 (30)		2 (17)		6 (40)		2 (25)	
**Obesity status, n (%)**									
	<Moderate obesity	30 (57)		9 (75)		10 (67)		4 (50)	
	≥Severe obesity	23 (43)		3 (25)		5 (33)		4 (50)	

^a^Cluster 1: high in principal component 1, high in principal component 2.

^b^Principal component 1: high or low adherence level; principal component 2: late or early behavior change.

^c^Cluster 2: low in principal component 1, high in principal component 2.

^d^Cluster 3: low in principal component 1, low in principal component 2.

^e^Cluster 4: high in principal component 1, low in principal component 2.

## Discussion

### Principal Findings

This study analyzed the effects of changes in lifestyle behaviors on weight loss during an obesity intervention, using behavioral dynamics in children and adolescents. In this process, behavioral phenotypes, including *high or low adherence level* and *late or early behavior change*, were identified by extracting PC factors from the functional data of individual children using FPCA. The *high or low adherence level* was associated with weight change for adherence to screen time, fruit and vegetable intake, exercise, drinking water, and sleep duration, whereas the *late or early behavior change* had a significant relationship with weight loss for changes in screen time, fruit and vegetable intake, and sleep duration.

The pattern of changes in the 5 main behavioral domains during the obesity intervention was identified using the FDA, and 2 representative behavioral phenotypes were derived from the individual change dynamics of each participant. The first was the *high or low adherence level,* wherein children with a higher score in the phenotype showed a high adherence to the behavioral goals at the beginning of the treatment, which was maintained until the end of the intervention. In contrast, for children with a lower phenotype score, the adherence rate was low at the beginning of treatment and remained low until the end of treatment. This representative phenotype of consistent change explained approximately 47% to 69% of each factor, which indicates that a large part of lifestyle behaviors was stable throughout the intervention.

According to the transtheoretical model of change, early behavioral change can be explained by differences in the stages of motivation to change. Children in the preparation and action phases at the beginning of an intervention may be ready to make changes in lifestyle behaviors, whereas children in the precontemplation and contemplation phases may be more resistant to change, even during participation in an obesity intervention [40]. The results of this study showed that a large part of the behavioral dynamics during the intervention could be explained by early compliance with changes. In other words, people behaved consistently throughout the intervention. Previous studies have also acknowledged that early behavioral compliance is a significant predictor of long-term compliance in alcohol abstinence [41], treatment of obstructive sleep apnea [42,43], and treatment of obsessive-compulsive disorder [44]. The results from this study add to the evidence for the importance of early behavioral changes in obesity treatment among children and adolescents.

The second behavioral phenotype derived from individual dynamics was the *late or early behavior change*. Children with a higher score in this phenotype, or *late behavior change*, showed a pattern of lower adherence to behavioral goals at the beginning of the intervention, which sharply increased after early the midtreatment period, and compliance was maintained until the end of the intervention. In contrast, children with low scores on the phenotype, or *early behavior change*, showed a high degree of compliance at the beginning of treatment; however, the behavioral change was not maintained and gradually decreased after 8 to 10 weeks. This phenotype explained approximately 11% to 17% of the entire dynamic phenotype, with a lower explanatory power than the first phenotype.

Children who appertain to the *late behavior change* phenotype may have progressed from the precontemplation or contemplation stages to the preparation or action stages of change during the treatment [45]. This behavior change may be associated with an increase in motivation toward change [46,47] as an effect of participating in the obesity intervention, with an emphasis on motivational interviewing strategies. Although most participants maintained their initial behavior, some of the behavioral dynamics can be explained by a change in behavior during the intervention, which supports the clinical relevance of conducting an obesity intervention aimed at changing behavior.

Several behavioral phenotypes were associated with weight change according to the regression model even after controlling for the effects of cross-sectional variables, which are known to be related to weight change. For adherence to using less screen time, both the *high adherence level* and *early behavior change* were associated with weight change, with a positive relationship with weight gain for the second phenotype. A possible explanation for this result is that for screen time, it may be important to maintain a high level of adherence at the beginning of an intervention. It is known that >2 hours of screen time per day is associated with obesity in children [48]. The results of this study add to the literature that efforts to reduce screen time, especially during the early stages of the intervention, may affect weight loss, even if the adherence rate decreases afterward.

For consuming fruits and vegetables, contrary to our expectations, the *low adherence level* was associated with weight gain. A possible explanation for this observation is that children with a higher score for fruit and vegetable consumption during the intervention process may have had a higher level of calorie intake, as reported in a previous meta-analysis [49]. Accordingly, a higher adherence to fruit and vegetable intake may have been associated with weight gain in this sample. Moreover, children with a higher score on the *late behavior change* showed a gradual increase in compliance with fruit and vegetable intake, which may reflect a pattern of change in the composition of the diet with a progressive increase in vegetable intake. In this study, we found that for the intake of fruits and vegetables, the *change* in the behavior was associated with weight loss, in contrast to the maintenance of the behavior.

For adherence to exercise and drinking water, only the *high adherence level* was associated with weight loss. The initial average scores for compliance with exercise and drinking water instead of sugar-sweetened beverages were 4.2 and 4 points, respectively, which were higher than the level of compliance with other lifestyle behaviors. Children who appertain to the *high adherence level* for exercise and drinking water persistently adhered to the lifestyle behavior from the beginning of the intervention and may even have maintained healthy lifestyle behaviors before the intervention. Several other studies have shown that physical activity and water intake are important lifestyle behaviors associated with weight loss [50,51]. This study is one of the first to show that it is important to maintain the level of adherence to physical activity and drinking water during an intervention for weight loss. In this regard, interventions based on self-determination theory and self-regulation theory may help maintain the initial behavior change, which claims that behavior that is congruent with an individual’s values is likely to be sustained [46,52].

For sleep duration, both the *high adherence level* and the *late behavior change* were associated with weight change. Similarly, a study showed that there were no differences between early abstainers and delayed abstainers for nonsmoking in adults [53]. Regardless of the time point of the behavioral change, children with obesity may benefit from adherence to sleep duration as long as sleep is maintained until the end of the intervention. Previous studies have found that sleep duration is associated with weight loss in children with obesity [54]. The findings of this study add to the literature in that the patterns of both early and delayed compliance explain weight loss according to sleep duration.

Finally, a cluster analysis to classify participants with similar characteristics revealed that most participants (53/88, 60%) were consistently adherent to each behavioral goal (+PC 1) and were more energetic toward the end (+PC 2). There were more children (39/53, 74%) in this cluster, indicating that younger participants were more likely to show an overall high level of adherence, especially toward the end of the intervention.

### Strengths

There are several benefits to understanding behavioral phenotypes from a dynamic perspective. It is difficult to comprehend the pattern of changes during the intervention process using the conventional method of comparing the data collected before the intervention and at the end point. For instance, the mean values of sleep duration were not significantly different at the beginning and end of the trial, whereas temporal dynamics indicated that there was a significant fluctuation during the intervention. Temporal dynamics showed that the maintenance of behavioral change was the major behavioral pattern during the intervention. To a lesser extent, some of the behavioral dynamics were explained through changes in behavior, indicating that some components of lifestyle behaviors can be changed by participating in an obesity intervention.

The findings of this study also imply that it is necessary to adopt different approaches depending on the stage of a child’s behavioral change. For children in the action stage, who are already prepared for behavior change at an early phase of the intervention, it may be more effective to work on specific behavior strategies to maintain the behavior in activation rather than enhancing motivation for behavior change [47]. Even if the level of adherence to behavioral goals is low at the beginning, behavioral changes attained in the early-middle stage of the intervention can lead to weight loss. Therefore, there is a need to enhance motivation among participants with lower motivation levels during the early phase of the intervention.

### Limitations

This study has several limitations that require cautious interpretation of the findings. First, the sample size was relatively small (n=88), after excluding variables with missing values. The study population was predominantly male (62/88, 71%). However, a statistical method that is robust to small sample sizes was used for this purpose [55]. Second, dynamic behavior variables were not measured over a large number of time points. In the future, it may be useful to analyze behavioral dynamics by measuring momentary information using digital methods such as ecological momentary assessment. Third, there may be important clinical relevance in identifying the characteristics of children and parents associated with behavioral phenotypes. This identification can be achieved using methods such as functional cluster analysis [38], which is a research question that should be addressed by future researchers. Finally, some of the demographic characteristics were significantly different between the participants included in the analysis and those who were excluded because of missing data. For example, baseline self-esteem, stages of change, exercise frequency, baseline adherence to drinking water, and sleep duration were lower in the exclusion group (Multimedia Appendix 1). This may be because those with missing data had lower motivation to participate in the intervention than participants with adequate data collected during the intervention. This requires cautious interpretation of the study findings, which could be relevant for participants with a higher level of participation in the intervention.

### Conclusions

In conclusion, a large part of the behavioral dynamics during the intervention was explained by the *high adherence level*, wherein children who made changes at the early stage of the intervention maintained the changed behaviors. A smaller part of the behavioral dynamic was explained through the *late or early behavior change*, where changes in behavior were attained after the early to midpoint of the intervention. For lifestyle behaviors associated with energy expenditure, such as exercise and screen time, earlier compliance to behavioral goals may be needed to expect weight loss. For other health-related behaviors, gradual improvement in compliance may also be helpful for weight management. As distinctive patterns of changes are associated with weight loss in lifestyle behaviors related to obesity, health care providers should carefully monitor the dynamics of compliance with these lifestyle behaviors during treatment. The findings of this study support the need for active interventions aimed at enhancing motivation to induce these changes in behavior.

## Appendix

Multimedia Appendix 1 Baseline characteristics of participants included versus excluded in the analysis.

## Abbreviations

FDA: functional data analysis
FPCA: functional principal component analysis
mHealth: mobile health
PC: principal component
