# Correction: Exploring the Effect of the Dynamics of Behavioral Phenotypes on Health Outcomes in an mHealth Intervention for Childhood Obesity: Longitudinal Observational Study

**DOI:** 10.2196/57013

**Published:** 2024-02-23

**Authors:** Sarah Woo, Sunho Jung, Hyunjung Lim, YoonMyung Kim, Kyung Hee Park

**Affiliations:** 1 Department of Medical Sciences, College of Medicine, Hallym University Chuncheon-si Republic of Korea; 2 School of Management, Kyung Hee University Seoul Republic of Korea; 3 Department of Medical Nutrition, Kyung Hee University Yongin-si Republic of Korea; 4 University College, Yonsei University International Campus Incheon Republic of Korea; 5 Department of Family Medicine Hallym University Sacred Heart Hospital Hallym University Anyang-si, Gyeonggi-do Republic of Korea

In “Exploring the Effect of the Dynamics of Behavioral Phenotypes on Health Outcomes in an mHealth Intervention for Childhood Obesity: Longitudinal Observational Study” (J Med Internet Res 2023;25:e45407) a figure number and some symbols were misnamed. The following corrections have been made:

In the second paragraph of the ‘FPCA Results’ subsection under the Results section, Figure 4 was misnamed as Figure 3. Changes were made in the following sentences.

The original passage:

In PC1, adherence to missions 1, 4, and 5 decreased at the end of the intervention (Figure 3A, 3G, and 3I), but compliance to missions 2 and 3 maintained high scores throughout the intervention (Figure 3C and 3E). Participants with –1 SD from the average had a low score at the beginning of the intervention and maintained a low level below average throughout the treatment.

has been changed to:

In PC1, adherence to missions 1, 4, and 5 decreased at the end of the intervention (Figure 4A, 4G, and 4I), but compliance to missions 2 and 3 maintained high scores throughout the intervention (Figure 4C and 4E). Participants with –1 SD from the average had a low score at the beginning of the intervention and maintained a low level below average throughout the treatment.

The following passage:

Participants with +1 SD from the average on PC 2 of behavioral phenotypes 1, 2, 3, and 5 showed a pattern of a low score at the beginning of the treatment, followed by a rapid increase at 8 to 10 weeks of the intervention (Figure 3B, 3D, 3F, and 3J).

was changed to:

Participants with +1 SD from the average on PC 2 of behavioral phenotypes 1, 2, 3, and 5 showed a pattern of a low score at the beginning of the treatment, followed by a rapid increase at 8 to 10 weeks of the intervention (Figure 4B, 4D, 4F, and 4J).

The following passage:

PC 2 of the fourth behavioral phenotype exhibited a slightly distinctive pattern, wherein participants with +1 SD showed a gradual increase throughout the intervention. In addition, the crossing point of participants with +1 SD and −1 SD was between 10 and 15 weeks of intervention, which is a later point compared with other phenotypes (Figure 3H).

has been changed to:

PC 2 of the fourth behavioral phenotype exhibited a slightly distinctive pattern, wherein participants with +1 SD showed a gradual increase throughout the intervention. In addition, the crossing point of participants with +1 SD and −1 SD was between 10 and 15 weeks of intervention, which is a later point compared with other phenotypes (Figure 4H).

In Figure 5, the seventh symbol from the left originally appeared as ‘M3 (PC2)’, and now reads ‘M4 (PC1)’. The eighth symbol originally appeared as ‘M4 (PC1)’ and now reads ‘M4 (PC2).

The correction will appear in the online version of the paper on the JMIR Publications website on February 23, 2024, together with the publication of this correction notice. Because this was made after submission to PubMed, PubMed Central, and other full-text repositories, the corrected article has also been resubmitted to those repositories.

**Figure 5:**
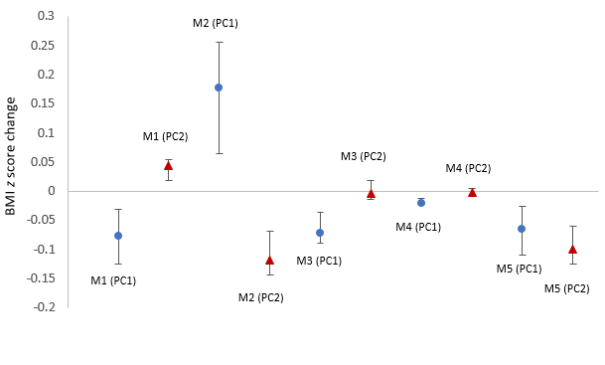
Effect of behavioral phenotypes on BMI z score change. PC: principal component. M1: screen time less than 2 hours; M2: eating more than 5 servings of fruits and vegetables; M3: exercising for more than 1 hour; M4: drinking water or plain milk; M5: sleeping for more than 8 hours; PC 1=high or low adherence level; PC 2=late or early behavioral change. Data were presented as standardized β coefficients and standard regression coefficients with 95% CIs. M: mission.

