# Supplementary material for: Exploring the Effect of the Dynamics of Behavioral Phenotypes on Health Outcomes in an mHealth Intervention for Childhood Obesity: Longitudinal Observational Study
Source: J Med Internet Res. 2023 Aug 17;25:e45407. doi: 10.2196/45407 (PMC10472181; doi:10.2196/45407)
Supplement: Multimedia Appendix 1 [file jmir_v25i1e45407_app1.docx]

Supplementary Table 1. Baseline characteristics of participants included vs. excluded in the analysis

| Variable | Included participants  (n=88) | Excluded participants (n=72) | ***p*** |
| --- | --- | --- | --- |
| Age (years) | 11.46±1.69 | 11.88±1.84 | 0.13 |
| Baseline BMI z-score | 2.60±0.90 | 2.67±0.87 | 0.61 |
| Monthly household income  (1,000,000 KRW) | 544.32±167.03 | 528.57±195.52 | 0.59 |
| Self-esteem | 37.38±7.68 | 34.94±6.91 | 0.04 |
| Internalizing problem | 54.89±11.24 | 55.65±11.20 | 0.67 |
| Externalizing problem | 53.89±10.43 | 54.73±10.85 | 0.62 |
| Stages of change | 3.43±0.77 | 3.14±0.97 | 0.04 |
| Beverage intake (frequency/week) | 2.05±1.20 | 2.33±1.16 | 0.13 |
| Vegetable intake (frequency/week) | 4.07±1.89 | 3.53±1.85 | 0.07 |
| Sleep duration (min/day) | 538.72±68.03 | 517.28±76.12 | 0.06 |
| Screen time (hours/day) | 3.92±1.18 | 4.10±1.08 | 0.33 |
| Exercise frequency (week) | 3.24±1.17 | 2.75±1.16 | 0.009 |
| Maternal BMI (kg/m2) | 25.00±4.37 | 24.00±3.01 | 0.12 |
| Paternal BMI (kg/m2) | 27.35±3.20 | 26.85±3.11 | 0.36 |
| Maternal psychosocial stress | 17.23±7.68 | 20.35±8.69 | 0.02 |
| Paternal psychosocial stress | 16.73±7.64 | 18.00±7.31 | 0.32 |
| Maternal positive parenting | 27.74±3.89 | 26.66±4.76 | 0.14 |
| Paternal positive parenting | 25.49±4.34 | 26.03±4.50 | 0.47 |
| Maternal negative parenting | 27.01±7.56 | 29.66±7.40 | 0.03 |
| Paternal negative parenting | 25.98±6.07 | 26.64±5.67 | 0.51 |
| Mission 1 (baseline) | 3.12±1.53 | 2.75±1.41 | 0.12 |
| Mission 2 (baseline) | 3.35±1.26 | 3.10±1.26 | 0.20 |
| Mission 3 (baseline) | 4.20±1.30 | 3.99±1.14 | 0.21 |
| Mission 4 (baseline) | 3.98±1.11 | 3.49±1.44 | 0.02 |
| Mission 5 (baseline) | 2.77±1.38 | 2.04±1.39 | 0.001 |

Abbreviations: BMI = body mass index; KRW = Korean Republic Won.

Parental psychosocial stress was measured using the Psychological Well-Being Index-Short Form (PWI-SF). Parental positive and negative parenting styles were measured with the Korean-Parents as Social Context Questionnaire (K-PSCQ).

Mission 1 = screen time less than two hours; Mission 2 = eating more than five servings of fruits and vegetables; Mission 3 = exercising for more than one hour; Mission 4 = drinking water or plain milk; Mission 5 = sleeping for more than eight hours.

Data are presented as mean ± SD with the p-value of the t-test for continuous variables.
